# Pathways to Age of Onset of Heroin Use: A Structural Model Approach Exploring the Relationship of the COMT Gene, Impulsivity and Childhood Trauma

**DOI:** 10.1371/journal.pone.0048735

**Published:** 2012-11-14

**Authors:** Ting Li, Jiang Du, Shunying Yu, Haifeng Jiang, Yingmei Fu, Dongxiang Wang, Haiming Sun, Hanhui Chen, Min Zhao

**Affiliations:** Shanghai Mental Health Center, Shanghai Jiao Tong University School of Medicine, Shanghai, China; Sapienza University of Rome, Italy

## Abstract

**Background:**

The interaction of the association of dopamine genes, impulsivity and childhood trauma with substance abuse remains unclear.

**Objectives:**

To clarify the impacts and the interactions of the Catechol -O-methyltransferase (COMT) gene, impulsivity and childhood trauma on the age of onset of heroin use among heroin dependent patients in China.

**Methods:**

202 male and 248 female inpatients who meet DSM-IV criteria of heroin dependence were enrolled. Impulsivity and childhood trauma were measured using BIS-11 (Barratt Impulsiveness Scale-11) and ETISR-SF (Early Trauma Inventory Self Report-Short Form). The single nucleotide polymorphism (SNP) rs737866 on the COMT gene-which has previously been associated with heroin abuse, was genotyped using a DNA sequence detection system. Structural equations model was used to assess the interaction paths between these factors and the age of onset of heroin use.

**Principal Findings:**

Chi-square test indicated the individuals with TT allele have earlier age of onset of heroin use than those with CT or CC allele. In the correlation analysis, the severity of childhood trauma was positively correlated to impulsive score, but both of them were negatively related to the age of onset of heroin use. In structure equation model, both the COMT gene and childhood trauma had impacts on the age of onset of heroin use directly or via impulsive personality.

**Conclusions:**

Our findings indicated that the COMT gene, impulsive personality traits and childhood trauma experience were interacted to impact the age of onset of heroin use, which play a critical role in the development of heroin dependence. The impact of environmental factor was greater than the COMT gene in the development of heroin dependence.

## Introduction

Substance abuse is a complex disease, which is the result of an interaction among various factors, such as genetic and environmental risk factors, and the personality of the individuals [1], [2]. Several studies have shown that genetic factors account for 30–60% of the risk for substance dependence [2], with most evidence involving the dopamine pathway, GABA and glutamatergic systems [3], [4]. Catechol -O-methyltransferase (COMT) catalyzes the degradation of dopamine, which plays critical role in the reinforcement mechanism of drug dependence. Our previous work indicated that the COMT gene SNP rs737866 was associated with heroin dependence. Individuals with the TT genotype would have an earlier age of onset of drug use than individuals with CT or CC genotypes. SNP rs737866 locates in the first intronic region of COMT, may in linkage disequilibrium with other functional SNPs and affect the transcription of COMT [5]. However, how the COMT SNP rs737866 interacted with other factors such as personality traits and childhood trauma in the development of opiate dependence is unclear. Some studies in the literature indicate that childhood trauma is a significant risk factor for substance abuse and other psychiatric disorders [6], [7]. Childhood trauma includes neglect, physical abuse, sexual abuse, and emotional abuse; all of which are risk factors for the development of substance abuse in later life [8]. However, the mechanism of how childhood trauma leads to the development of substance abuse is unclear.

Other studies have focused on the impulsive personality of substance abusers and Moeller et al have found that the most marked difference in characteristics between substance users and control subjects is a high level of impulsivity in substance users [9]. Impulsivity was also found to be a high-risk factor for early substance use, and related to the severity of drug abuse and treatment retention [10], [11]. Moreover, some studies have indicated that impulsivity were significant positively correlated to the total number of illicit drugs used in lifetime, while, the age of first substance use was negatively correlated to impulsivity score [12].

There are much evidence to show that impulsivity is related to the underlying biological and psychosocial processes. For example, linkage and association studies have indicated that polymorphisms in COMT, Dopamine D4 receptor and Dopamine Transporter (DAT) gene are associated with abnormally aggressive behavior or personality disorders [2]. Animal studies have also demonstrated the key role of genes such as COMT and 5HTR2B, in mediating impulsive behavior [13], [14]. Meanwhile, within the psychological literature, impulsive personality has been thought be linked with early childhood experience of trauma and loss. A cross-sectional study in Brazil indicated that childhood trauma was strongly associated with executive dysfunction and impulsivity [15].

Given the current evidence, we predict that the interaction of COMT gene, childhood trauma and impulsive personality contribute to the development of heroin abuse. Indeed, there are few studies which looked at the relationship among these factors. The respective influence of COMT gene, childhood trauma and impulsivity personality in the development of heroin dependence, is also unknown. In the study, we try to explore how the COMT gene, severity of childhood trauma and impulsive personality interacted to influence the age of onset of heroin use and the respective degree of impact for the three factors.

## Materials and Methods

### Ethics Statement

This research protocol was approved by the Ethic Committee of the Shanghai Mental Health Center. Subjects were recruited by the research team who were trained to provide confidentiality. According to the related law in China, the patients in drug rehabilitation center have the equal civil rights of citizens except they are required to accept the drug rehabilitation for a certain period of time according the severity of their drug dependence. In order to minimize the possible coercion, the researchers interacted with the potential participants individually without the police officer present, and explain to the potential subjects that their participation will not result any consequences or benefits to them while in the rehabilitation center, and they may decline to participate or withdraw from the study at any time. Only after it was certainly that the participant understood and agreed, the informed consent was signed. The study participants were given a copy of the consent form and the status of inform consent was not needed to notify the policy office in the rehabilitation center.

### Subjects

A total of 450 currently abstinent patients who had previously been dependent on heroin were recruited from drug rehabilitation centers in Shanghai, a large metropolitan city in Eastern China, which included 202 men and 248 women with a mean (SD) age of 33.9 (7.7) years. A diagnosis of heroin dependence according to the fourth edition of the Diagnostic and Statistical Manual of Mental Disorders (DSM-IV) was made during a psychiatric interview using structured clinical interview for DSM-IV (SCID-I). Subjects with other axis I psychiatric diagnoses were excluded from the study. A self-completion form developed for the study which included basic demographic information and the history of drug use (onset age of drug use, frequency and amount of current daily use, other drug use, times of previous drug treatment, etc.) was collected. Urine and blood samples were obtained and screened for illicit drugs and genotype.

### Measures

**1.** The Barratt Impulsiveness Scale (BIS-11). The BIS-11 is a 30 item self-report scale that is structured to assess aspects of impulsivity relating to rash behavior or thoughts by asking the respondents to answer questions about the ways they act and think without reference to any specific period [16]. The BIS-11 has a total score and contains three subscales. The first scale measures motor impulsiveness (BIS-M) by assessing fast reactions and/or restlessness, the second measures attention impulsivity (BIS-A) by assessing problems related to concentrating or paying attention and the third scale evaluates non-planning impulsivity (BIS-NP) toward planning and thinking carefully, or enjoy challenging mental tasks. The BIS-11′s internal consistency coefficients range from 0.79 to 0.83 for different populations [16]. Its reliability and validity has repeatedly been proved in a variety of languages [17], [18], [19].

**2.** Early Trauma Inventory Self Report-Short Form (ETISR-SF). The ETISR-SF evaluates trauma across four subscales, including general trauma, physical abuse, emotional abuse, and sexual abuse. Higher scores in each domain indicate more traumatic experiences [20]. It has good validity and reliability, with the Cronbach’ alphas for these 4 domains were ranging from 0.7 to 0.87 [21]. The Chinese version of ETISR-SF showed a similar range of Cronbach’s alpha values from 0.67 to 0.84 [22].

### SNP Selection and Genotyping for COMT Gene

Genomic DNA for each subject was extracted from lymphocytes according to a modified phenol/chloroform method. The COMT gene SNP rs737866 was genotyped on the ABI Prism 7900 sequence detection system. For quality control, 5% of random DNA samples were genotyped twice for each SNP to calculate genotyping error. The genotyping accuracy was 100%.

### Statistical Analyses

**Figure 1:**
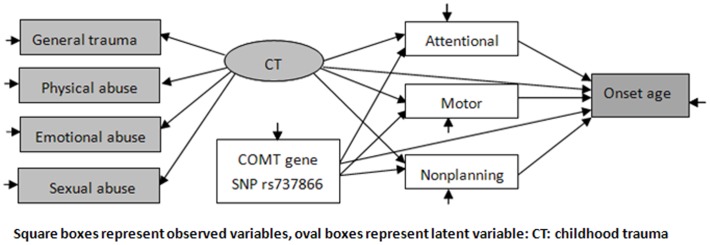
Baseline structural model.

Independent t- tests were used to compare the mean NS subscale scores and the mean onset age of heroin use among groups defined according to different COMT gene SNPs genotypes. Structural equations model is a generalization of linear regression and factor analysis models; it is used for the multivariate analysis. We used a model (see Figure 1 for the baseline structural equations model) to describe the interaction paths among COMT gene SNP rs737866, childhood trauma and the personality of impulsivity. The model was constructed according to the interaction of the gene and the environment. We hypothesized that the relationships between different exogenous variables exploring childhood trauma (general trauma, physical abuse, emotional abuse, sexual abuse) can be explained by the presence of an underlying latent variable, labeled CT, child trauma. Impulsive personality was represented by three variables: attentional, motor, and non-planning. In these models, the COMT gene SNP rs737866 and childhood trauma could influence the age of onset of heroin use directly or indirectly through impulsivity. Correlations between these variables were also computed. For the present study, coefficients below 0.10 were considered as negligible (and represented with dotted arrows) even if they were statistically significant.

There are several parameters that were selected: Steiger’s RMSEA (root mean square error approximation), where a value smaller than 0.1 is considered as a good fit and very good when it is smaller than 0.05, Bentler and Bonnet’s NFI (normed fit index) where a value over 0.95 is considered as a good fit and Akaike’s information criterion (AIC). AIC adopts a balanced approach taking into account the quality of the model, and it discourages “over-fitting”. Statistical power was computed with a null assumption of good fit corresponding to an RMSEA of 0.05 and an alternative assumption of bad fit for an RMSEA equal to or higher than 0.1. A power higher than 90% was obtained. All the tests were performed with a = 5%. For statistical analyses, SPSS15.0 and Amos 7.0 software were used for the structural equations models.

## Results

### Demographic and Drug Use Data

Demographic and drug use history of the subjects is summarized in Table 1. Genotype and allele frequencies of the COMT gene SNP rs737866 in heroin dependence patients were shown in Table 2, and they were in Hardy-Weinberg equilibrium (χ2 = 1.14; P = 0.285).

**Table 1:** Main demographic characteristics and drug use history.

Covariates	N = 450
Mean(SD) of education	10.3(2.0)
Marriage status	
*Married, n(%)*	127(28.2)
*Divorced or separated n (%)*	99(20.0)
*Unmarried n (%)*	224(49.8)
Mean onset age of drug use (years)	22.9(6.0)
Mean duration of drug use (years)	10.9(4.4)
Mean daily intake of Heroin in gram	0.97(0.6)
Mean daily frequency of drug use	4.10(1.7)
Number of episodes of treatment forsubstance dependence	6.97(7.9)
BIS-11 Total score	66.6(8.5)
*Motor*	24.1(4.30)
*Non-planning*	24.6(4.31)
*Attentional*	17.9(2.75)
ETISR-SF	
*General trauma*	1.09(1.50)
*Physical abuse*	0.68(0.94)
*Emotional abuse*	0.93(1.81)
*Sexual abuse*	0.39(0.93)

**Table 2:** The genotype distribution of COMT SNP rs737866.

SNP	Genotype			Gene	
rs737866	CC	CT	TT	C	T
(n = 450)	39(8.7)	203(45.1)	208(46.2)	162(31.2)	619(68.2)

### The Relationship Among COMT Gene SNP rs737866, Onset Age of Drug Use and the Subscale of BIS and ETISR-SF

Based on our previous study, the COMT rs737866 SNP genotype was re-coded as a dichotomous variable for the recessive model (1 = “TT”, 2 = “CT” or “CC”). The individuals with TT allele have earlier age of onset of heroin use than whom with CT or CC allele (t = 3.51; P<0.001). Pearson correlation analysis showed the BIS total score was negatively related to the age of onset of heroin use(r = −0.106, P = 0.024).The ETISR-SF score was positively correlated to impulsive score (r = 0.120, P = 0.011), but negatively correlated to the age of onset of heroin use(r = −0.278, P<0.0001).

### Structural Equations Models of Pathways to Substance Use

**Figure 2:**
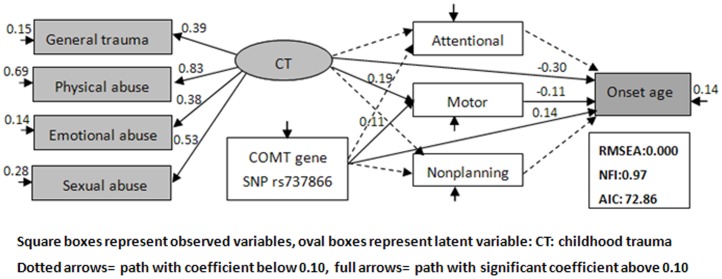
Pathways to the age onset of heroin use in heroin dependent patients.

As showed in Figure 2. Our model yielded a very good fit, and the RMSEA estimate was 0.000, Bentler and Bonett’s NFI was 0.967. Chi-square test was not rejected with χ2 = 16.86, df = 17, P = 0.464. Akaike information criterion (AIC) was 72.86. All the results showed an excellent match between the models and the data.

All coefficients, except Attentional and Nonplanning, were significant in the model, varying from 0.11 to 0.30. The coefficients associated with the latent variable CT were statistically significant, ranging from 0.38 to 0.83, were in favor of its robustness.

### Influence of CT and Impulsivity (Environmentally Driven Factors)

As showed in Figure 2. For the model, CT had a significant influence on the age of onset of heroin use (−0.30). CT also had a moderately significant indirect influence via the motor factor- one aspect of impulsive personality (−0.02) on the age of the age of heroin onset use, and CT had a slight influence on the motor factor (0.19). So the interpretation was straightforward, the more serious the childhood trauma, the individuals would have more impulsiveness, and begin to use heroin earlier.

### Influence of COMT Gene SNP (Biologically Driven Factors)

In this model, the direct influence of the COMT gene SNP on the age of onset of drug use was significant (−0.14), that is, individuals with TT genotype were more likely begin drug use earlier. There was also moderately significant indirect influence of the COMT gene SNP rs737866 through impulsive personality (0.01). In turn, motor impulsive personality was also influenced by COMT gene SNP rs737866; The coefficient was 0.11.

## Discussion

This study highlights several points:

Firstly, we observed that differences in the severity of childhood trauma and the variation of the COMT gene SNP rs737866 was associated with the development of drug use. Although both of these factors influenced the age of onset of drug use, childhood trauma appeared to be the more influential than the COMT gene.

Gene and environmental interactions on psychiatric diseases have been widely accepted and our study suggests that there is an interaction between the COMT gene and childhood trauma in the development of heroin use. This is in line with previous literature that both of these risk factors may interact to contribute to the occurrence of psychiatric disease. Specifically, Savitz *et a.l* suggested that COMT may interact with childhood trauma to impact the risk of developing schizotypal personality traits [23]. Perroud *et al.* showed that the COMT gene modulates the influence of childhood abuse on anger traits, which was associated with eating disorder and borderline personality disorder [24]. Wanger S *et al.* indicated that the COMT gene modulated the association of serious life events and impulsive aggression in female patients with borderline personality disorder [25]. We propose that the impact of childhood trauma on the age of onset of heroin use is stronger than that of the COMT gene, indicating the importance of the role of the environment.

Secondly, impulsive personality was also found to play a role in the onset of heroin use. Both childhood trauma and the COMT gene were associated with the age of onset of drug use through impulsive personality. Among the three factors, the motor factor appeared to have the largest effect on the age of onset of drug use.

According to the theory, which stipulates that personality is influenced by environment and genes, we observed that impulsive personality was significant correlated to childhood trauma and the COMT gene. Among the three factors, motor factor had the strongest influence on the onset of drug use. Motor factor was defined as “acting on the spur of the moment” by Patton [16], that was, the more serious the childhood trauma, the greater the motor activation and the earlier the age of drug use. This was consistent with literature that multi-factor impacts the development of substance abuse, including the reciprocal relationship between impulsivity and environmental influences, such as childhood trauma and poor parenting [26]. Specifically, some literature suggested that childhood trauma would lead many adolescents run away from home, causing high rates of continued traumatic stress, self-harm and substance use [27], [28], [29].

There are some limitations need to be considered. Our structural equation model only partially took into consideration of a limited number of variables, so we could not rule out other possible factors which may influence the age of onset of drug use. Moreover, our model only focused one behavior phenotype, that is the age of onset of drug use, we could not be sure that the interaction have underline other behavior phenotype, which may also influence the development of drug use disorders. Now we are investigating if the result is reproducible for other behavior phenotype.

Despite these limitations, the study provides new insights into the complex relationship between biological and environmental factors in the development of mental disorders. Moreover, the structural equation model is a neat way to explore the respective influence of different factors in the development of complex disorders. In this study, childhood trauma is the main risk factor underlying heroin use while environmental and genetic factors took into account.
